# Risk of metachronous gastric neoplasm occurrence during intermediate-term follow-up period after endoscopic submucosal dissection for gastric dysplasia

**DOI:** 10.1038/s41598-020-63722-0

**Published:** 2020-04-21

**Authors:** Young-Il Kim, Jae Yong Park, Beom Jin Kim, Hye Won Hwang, Soon Auck Hong, Jae Gyu Kim

**Affiliations:** 1 Center for Gastric Cancer, National Cancer Center, Goyang, Korea; 2 Graduate School of Medicine, Chung-Ang University, Seoul, Korea; 3 Department of Internal Medicine, Chung-Ang University College of Medicine, Seoul, Korea; 4 Department of Pathology, Chung-Ang University College of Medicine, Seoul, Korea

**Keywords:** Gastric cancer, Outcomes research

## Abstract

After endoscopic resection (ER) of gastric dysplasia, metachronous gastric neoplasm (MGN) appears to have an incidence rate similar to that detected after ER of early gastric cancer (EGC). We investigated whether the risk of MGN after ER for gastric dysplasia is different between patients with low-grade dysplasia (LGD) and high-grade dysplasia (HGD). Between March 2011 and December 2016, 198 patients with LGD (LGD group) and 46 patients with HGD (HGD group) who underwent ER were included in the study. During a median follow-up of 2.5 years, MGNs developed in 21 patients (10.6%) in the LGD group and in 6 patients (13.0%) in the HGD group. Hazard ratios (HRs) for MGNs (HR, 1.45; *P* = 0.425) and for metachronous HGD or gastric cancer (HR, 2.41; *P* = 0.214) in the HGD group were not different than those of the LGD group. However, considering patients without *Helicobacter pylori* infection, those in the HGD group had a significantly increased risk of metachronous HGD or gastric cancer compared to those in the LGD group (HR in HGD-group, 5.23; *P* = 0.044). These results indicate that meticulous surveillance endoscopy is needed to detect MGNs after ER of gastric dysplasia, especially in patients with HGD, including those without *H. pylori* infection.

## Introduction

Gastric cancer remains a major health concern and was the third leading cause of cancer death worldwide in 2018^1^. The presence of premalignant lesions is an important risk factor for the development of gastric cancer^2^. Among premalignant lesions, gastric dysplasia is a neoplastic lesion and the last stage in gastric carcinogenesis, especially the intestinal type^2^. Rate of progression from gastric dysplasia to invasive carcinoma was reported to be 2.8–11.5% in patients with low-grade dysplasia (LGD)^3–6^, and 10–68.8% in those with high-grade dysplasia (HGD)^3,6,7^. Long-term follow-up cohort studies showed a significant association between gastric dysplasia and an increased incidence of gastric cancer^6,8^.

Guidelines recommended endoscopic resection (ER) in patients with gastric dysplasia due to the increased probability of coexisting invasive carcinoma or the risk of progression^9–11^. Similar to early gastric cancer (EGC) patients who underwent ER, metachronous gastric neoplasms occurred in 12.1–14.6% patients with gastric dysplasia^12–14^. Thus, long-term regular follow-up might be needed after ER of gastric dysplasia. Few studies reported that the incidence of metachronous gastric neoplasms (MGNs) after ER of gastric dysplasia was comparable to that after ER of EGC^15,16^. Thus, these studies suggested long-term surveillance after ER of gastric dysplasia similar to the recommendation for surveillance after ER of EGCs^15,16^. However, the risk of MGNs after ER of gastric dysplasia might be different with respect to histologic grades (LGD vs. HGD) due to the different risks for gastric cancer associated with each grade.

In this study, we investigated whether the risk of MGN occurrence after ER in patients with LGD was different from that in patients with HGD.

## Patients and methods

### Patients

We retrospectively collected clinical and pathological data of consecutive patients who underwent endoscopic submucosal dissection (ESD) for gastric neoplasms at the Chung-Ang University Hospital between March 2011 and December 2016. The inclusion criteria were as follows: patients diagnosed with gastric dysplasia (LGD or HGD) on final pathological evaluations after ESD and patients with follow-up periods of more than 1 year. Patients were excluded if follow-up periods after ESD were less than 1 year, or if ESD was performed for EGC. This study was approved by the Institutional Review Board of Chung-Ang University Hospital (IRB number: 1801-002-16134). The requirement for informed consent from all included patients was waived by the Institutional Review Board due to the minimal risk of the study. The study was conducted in accordance with the Helsinki Declaration.

The patients’ clinical data that was analyzed included the following: baseline demographics, co-morbid diseases, and *Helicobacter pylori* infection status. Pathological data included the following: the presence of multiple initial lesions, tumor location, size, and histological grade of dysplasia.

### ESD procedures and follow-up schedule

Detailed ESD procedures were described in a previous study^17^. All ESD procedures were performed by experienced gastroenterologists who were certified specialty board members of the Korean Society of Gastrointestinal Endoscopy. After indigocarmine was applied to the dysplastic lesion, we made markings around the margin of lesion using electrocautery or argon plasma coagulation. A mixture of hyaluronic acid and/or normal saline, 0.2% indigocarmine, and 1:10,000 epinephrine solution was injected into the submucosa. Circumferential pre-cutting and submucosal dissection were performed using a needle knife, insulation-tipped knife, or a hook knife. After ESD, all patients underwent follow-up endoscopic examinations at 3 months, 12 months, and annually thereafter. At follow-up examinations, endoscopic biopsies were performed on the ESD scar as well as on all suspicious mucosal lesions.

### Pathological evaluation of gastric neoplasm, background atrophy and intestinal metaplasia

The Vienna classification was used for the diagnosis of gastric neoplasm^18^. In our study, category 3 (non-invasive low-grade adenoma/dysplasia) was considered as LGD, and category 4.1 (high-grade adenoma/dysplasia), and 4.2 (non-invasive carcinoma [carcinoma *in situ*]) were considered as HGD. Gastric cancer included category 4.3 (suspicion of invasive carcinoma) and category 5 (invasive neoplasia). Background atrophy and intestinal metaplasia status was assessed in the normal mucosa around the dysplasia lesion of resected ESD specimen, retrospectively. According to the updated Sydney system, atrophy and intestinal metaplasia were graded as absent, mild, moderate, or marked^19^. All pathological evaluations were performed by two experienced gastrointestinal pathologists.

### *H. pylori* infection status evaluation and treatment

At the time of gastric dysplasia diagnosis, *H. pylori* infection status was evaluated using a rapid urease test or by histology (Wright-Giemsa staining of biopsy specimen). Treatment for *H. pylori* infection was prescribed only in patients who chose to receive the treatment after ESD because the treatment costs were not covered by the Korean National Health Insurance. *H. pylori* infection status was negative according to the following criteria: (1) negative result of a urea breath test or (2) negative results in both rapid urease test and histology.

### Study outcomes

The primary outcome was the incidence of MGNs (LGD, HGD, and gastric cancer) detected at the 1-year follow-up or later. Secondary outcomes included the risk of metachronous HGD or gastric cancer detected at the 1-year follow-up or later, as well as factors associated with total MGNs and metachronous HGD or gastric cancers.

### Statistical analysis

The chi-square test or Fisher’s exact test were performed to compare categorical variables. For the comparison of non-categorical variables, an independent *t*-test or Mann-Whitney U test was performed. To evaluate the risk of total MGN occurrence and metachronous HGD or gastric cancer occurrence based on the grade of gastric dysplasia, we used the Kaplan-Meier method for estimating the incidence curves and Cox-proportional hazard regression models for estimating hazard ratios [HRs] and 95% confidence intervals [CIs]. Univariate and multivariate Cox-proportional hazard regression analyses were performed to investigate the risk factors associated with total MGNs and metachronous HGD or gastric cancer. All statistical analyses were performed using STATA 13.1 software (StataCorp, College Station, Texas, USA). *P* values < 0.05 were considered to be statistically significant.

## Results

### Baseline characteristics

**Figure 1:**
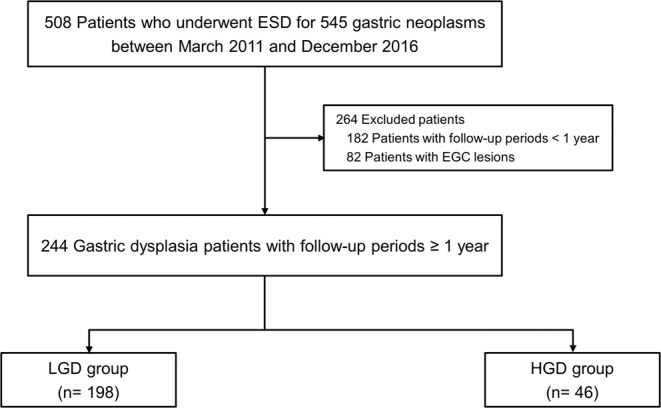
Flowchart of the study. EGC, early gastric cancer; ESD, endoscopic submucosal dissection; HGD, high-grade dysplasia; LGD, low-grade dysplasia.

In 508 patients who underwent ESD for 545 gastric neoplastic lesions, 244 patients with gastric dysplasia with follow-up durations longer than 1 year were included in the final analyses, in which 98 patients were diagnosed with low-grade dysplasia (LGD group) and 46 patients with high-grade dysplasia (HGD group) (Fig. 1).

Detailed baseline clinical and pathological characteristics are described in Table 1. The median age of the patients included in the study was 66 years (interquartile range [IQR], 58–71 years), and 61.1% of them were male. Baseline characteristics, including age, sex, body mass index, smoking, comorbidities, antiplatelet drug use, and distributions of background atrophy and intestinal metaplasia were not different between the LGD group and HGD group. The HGD group had significantly larger tumor size (mean tumor size, 1.6 cm vs. 1.1 cm; *P* = 0.001), and shorter follow-up duration (median 2.2 years vs. 2.7 years; *P* = 0.037), than those of the LGD group (Table 1).

**Table 1:** Baseline characteristics of patients with gastric dysplasia.

	LGD group (n = 198)	HGD group (n = 46)	*P*
Age (year), median (IQR)	66 (57–70)	66 (60–75)	0.248
Sex, no (%)			0.099
Male	116 (58.6)	33 (71.7)	
Female	82 (41.4)	13 (28.3)	
Body mass index (kg/m^2^), mean ± SD	24.8 ± 3.3	24.3 ± 3.1	0.177
Smoking, no (%)	64 (32.3)	17 (37.0)	0.548
Alcohol drinking, no (%)	73 (36.9)	17 (37.0)	0.991
Familial history of gastric cancer, no (%)	24 (12.1)	4 (8.7)	0.511
**Comorbidity, no (%)**			
Hypertension	77 (38.9)	20 (43.5)	0.567
Diabetes mellitus	43 (21.7)	6 (13.0)	0.186
Chronic liver disease	13 (6.6)	3 (6.5)	0.991
Chronic lung disease	13 (6.6)	2 (4.4)	0.743
Cardiovascular disease	15 (7.6)	4 (8.7)	0.763
Cerebrovascular disease	3 (1.5)	1 (2.2)	0.569
Other organ cancer	12 (6.1)	4 (8.7)	0.512
Antiplatelet drug use, no (%)	43 (21.7)	11 (23.9)	0.747
*H. pylori* status at baseline,* no (%)			0.93
Negative	90 (68.7)	19 (67.9)	
Positive	41 (31.3)	9 (32.1)	
*H. pylori* treatment after ESD,^†^ no (%)			0.135
No	130 (73.9)	30 (85.7)	
Yes	46 (26.1)	5 (14.3)	
Tumor size (cm), mean ± SD	1.1 ± 0.7	1.6 ± 0.9	0.001
Tumor location, no (%)			0.099
Lower third	141 (71.2)	26 (56.5)	
Middle third	44 (22.2)	14 (30.4)	
Upper third	13 (6.6)	6 (13.0)	
Multiple lesions, no (%)	13 (6.6)	5 (10.9)	0.346
Background atrophy, no (%)			0.666
Absent	34 (17.2)	5 (10.9)	
Mild	61 (30.8)	16 (34.8)	
Moderate	73 (36.9)	16 (34.8)	
Marked	30 (15.2)	9 (19.6)	
Background intestinal metaplasia, no (%)			0.497
Absent	11 (5.6)	5 (10.9)	
Mild	54 (27.3)	13 (28.3)	
Moderate	98 (49.5)	19 (41.3)	
Marked	35 (17.7)	9 (19.6)	
Follow-up duration after ESD (year), median (IQR)	2.7 (1.8–4.1)	2.2 (1.4–3.3)	0.037

ESD, endoscopic submucosal dissection; HGD, high-grade dysplasia; IQR, interquartile range; LGD, low-grade dysplasia; SD, standard deviation.

^*^The number of patients who had *H. pylori* status evaluations at baseline was 131 in the LGD group and 28 in the HGD group.

^†^The number of patients who had *H. pylori* treatment information after ESD was 176 in the LGD group and 35 in the HGD group.

### Risk of MGNs occurrence

**Figure 2:**
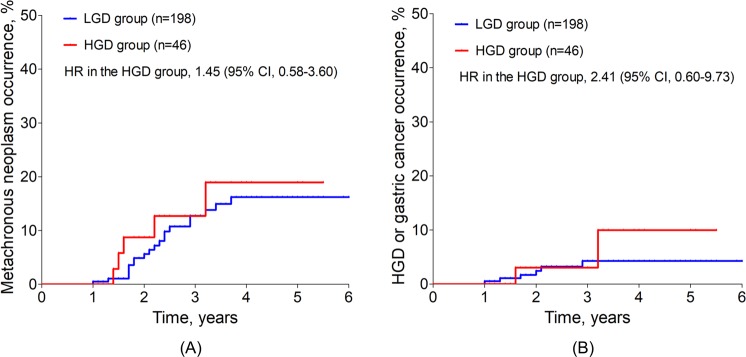
Risk of metachronous gastric neoplasm occurrences after ESD for gastric dysplasia based on histologic types. ESD, endoscopic submucosal dissection; HGD, high-grade dysplasia; LGD, low-grade dysplasia; HR, hazard ratio; CI, confidence interval. (**A**) Risk of metachronous gastric neoplasm in the HGD group (HR, 1.45; 95% CI, 0.58–3.60) was not different compared with the LGD group (HR, 1.00). (**B**) Risk of metachronous HGD or gastric cancer was not different between the LGD group (HR, 1.00) and the HGD group (HR, 2.41; 95% CI, 0.60–9.73).

During a median  follow-up period of 2.5 years (IQR, 1.8–4.0 years), MGNs were detected in 21 of 198 patients (10.6%, 36.3 cases/1,000 person-years) in the LGD group and in 6 of 48 patients (13.0%, 26.1 cases/1,000 person-years) in the HGD group (HR in the HGD group, 1.45; 95% CI, 0.58–3.60; *P* = 0.425). No patients developed synchronous gastric neoplasm after initial ESD. The risk of MGN occurrence was not significantly different between the two groups (Fig. 2A).

In 27 metachronous neoplasms, 9 neoplastic lesions were HGD (6 cases) or EGC (3 cases); proportions of metachronous HGD or EGC were 28.6% (6 in 21 MGNs) in the LGD group and 50.0% (3 in 6 MGNs) in the HGD group. Metachronous HGD or EGC occurred in six patients in the LGD group (3.0%) and in three patients in the HGD group (6.5%), and the incidence was not different between the two groups (HR in the HGD group, 2.42; 95% CI, 0.60–9.73; *P* = 0.214) (Fig. 2B).

### Treatment and risk factors associated with MGNs

**Figure 3:**
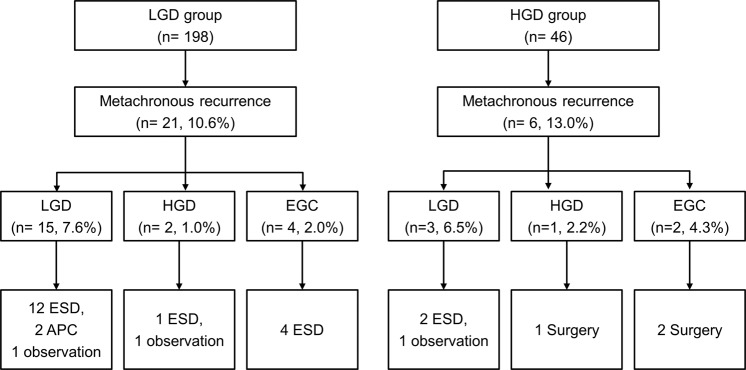
Follow-up after ESD for gastric dysplasia and management of metachronous gastric neoplasm. APC, argon plasma coagulation; EGC, early gastric cancer; ESD, endoscopic submucosal dissection; HGD, high-grade dysplasia; LGD, low-grade dysplasia.

The most common treatment modality for MGNs was ESD, which was performed to treat 19 of the 27 lesions (70.4%). Surgery was performed in three patients in the HGD group (two EGCs that did not meet the ESD indication and one HGD that remained after ESD and additional argon plasma coagulation). Two patients with metachronous LGD and one patient with metachronous HGD were carefully observed with regular follow-up endoscopic examinations without treatment due to the patient’s poor general condition or patient’s refusal to accept treatment (Fig. 3).

In comparing the clinical characteristics between patients who developed MGNs and those who did not, the characteristics were not different between both patient groups (Table 2). In addition, there were no significant clinicopathological factors including background atrophy and intestinal metaplasia grades associated with the development of MGNs and metachronous HGD or gastric cancer after ESD of gastric dysplasia in the Cox-proportional hazard regression analyses (Table 3 and Supplementary Table 1).

**Table 2:** Comparison of the clinical characteristics between patients with and without metachronous gastric neoplasm.

	Metachronous gastric neoplasm		*P*
	No (n = 217)	Yes (n = 27)	
Age (year), median (IQR)	66 (58–71)	66 (60–72)	0.616
Male sex, no (%)	131 (60.4)	18 (66.7)	0.527
Smoking, no (%)	39 (18.0)	3 (11.1)	0.373
Alcohol, no (%)	81 (37.3)	9 (33.3)	0.685
Antiplatelet drug use, no (%)	24 (11.6)	4 (14.8)	0.564
Familial history of gastric cancer, no (%)	49 (22.6)	5 (18.5)	0.632
*H. Pylori* status at last follow-up,* no (%)			0.827
Negative	190 (95.0)	24 (96.0)	
Positive	10 (5.0)	1 (4.0)	
Histologic type of dysplasia, no (%)			0.635
Low-grade dysplasia	177 (81.6)	21 (77.8)	
High-grade dysplasia	40 (18.4)	6 (22.2)	
Tumor size (cm), mean ± SD	1.2 ± 0.8	1.3 ± 0.9	0.366
Tumor location, no (%)			0.797
Lower third	147 (67.7)	20 (74.1)	
Middle third	52 (24.0)	6 (22.2)	
Upper third	18 (8.3)	1 (3.7)	
Initial multiple lesions, no (%)	16 (7.4)	2 (7.4)	0.995
Background atrophy, no (%)			0.439
Absent	37 (17.1)	2 (7.4)	
Mild	68 (31.3)	9 (33.3)	
Moderate	76 (35.0)	13 (48.1)	
Marked	36 (16.6)	3 (11.1)	
Background intestinal metaplasia, no (%)			0.428
Absent	16 (7.4)	0 (0)	
Mild	60 (27.6)	7 (25.9)	
Moderate	104 (47.9)	13 (48.1)	
Marked	37 (17.1)	7 (25.9)	

IQR, interquartile range; SD, standard deviation.

*The information of *H. pylori* status at the last follow-up was not available in 19 patients.

**Table 3:** Risk factors for metachronous gastric neoplasm.

	Univariate analysis*		*P*
	HR	95% CI	
Age, years	1.03	0.98–1.07	0.215
**Sex**			
Female	1.00		
Male	1.27	0.57–2.83	0.561
**Smoking**			
No	1.00		
Yes	0.40	0.12–1.35	0.139
**Alcohol**			
No	1.00		
Yes	0.75	0.34–1.69	0.492
**Antiplatelet drug use**			
No	1.00		
Yes	0.93	0.35–2.49	0.892
***H. Pylori*** **status at last follow-up**			
Negative	1.00		
Positive	1.01	0.14–7.51	0.992
**Histologic type of dysplasia**			
Low-grade dysplasia	1.00		
High-grade dysplasia	1.45	0.58–3.60	0.425
**Tumor size**			
<1.5 cm	1.00		
≥1.5 cm	1.22	0.54–2.72	0.635
**Tumor location**			
Lower third	1.00		
Middle third	0.95	0.38–2.40	0.919
Upper third	0.65	0.09–4.89	0.677
**Initial multiple lesions**			
No	1.00		
Yes	0.83	0.19–3.57	0.804
**Background atrophy**			
Absent	1.00		
Mild	2.40	0.57–10.08	0.231
Moderate to marked	3.33	0.85–13.13	0.085
**Background intestinal metaplasia**			
Absent	1.00		
Mild	3.78	0.20–72.62	0.377
Moderate to marked	5.13	0.28–92.51	0.268

HR, hazard ratio; CI, confidence interval.

^*^The Cox-proportional hazard regression model was used.

### Analysis of data from patients without *H. pylori* infection

**Figure 4:**
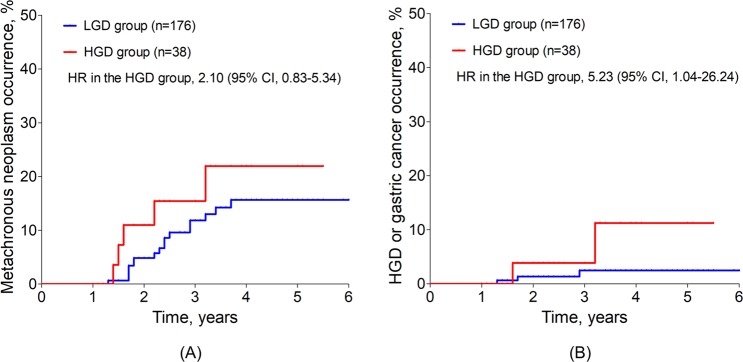
Risk of metachronous gastric neoplasm occurrences after ESD for gastric dysplasia based on histologic types in patients without *H. pylori* infection. ESD, endoscopic submucosal dissection; HGD, high-grade dysplasia; LGD, low-grade dysplasia; HR, hazard ratio; CI, confidence interval. (**A**) Risk of metachronous HGD or gastric cancer was not different between the LGD group (HR, 1.00) and the HGD group (HR, 2.10; 95% CI, 0.83–5.34). (**B**) Risk of metachronous HGD or gastric cancer in the HGD group (HR, 5.23; 95% CI, 1.04–26.24) was significantly higher compared to the LGD group (HR, 1.00).

At the time of gastric dysplasia diagnosis, 159 patients had a result of *H. pylori* infection status, and 68.6% of the patients (109/159 patients) were negative *H. pylori* infection. However, at the last follow-up, most included patients (95.1%, 214 of the 225 patients) did not have *H. pylori* infection because we treated *H. pylori* infection after ESD, if patients accepted treatment for *H. pylori* eradication (Table 1). Thus, we further analyzed the risks of MGNs and metachronous HGD or gastric cancer occurrences in 214 patients without *H. pylori* infection at follow-up. The MGNs were detected in 18 patients in the LGD group (34.3 cases/1,000 person-years) and in 6 patients in the HGD group (63.2 cases/1,000 person-years). Compared to the LGD group, the HGD group did not show a significantly increased risk for the development of MGNs (HR in the HGD group, 1.45; 95% CI, 0.58–3.60; *P* = 0.192) (Fig. 4A). Incidences of metachronous HGD or gastric cancer were 5.6 cases/1,000 person-years for the LGD group and 31.3 cases/1,000 person-years for the HGD group. The HGD group had a significantly higher risk of metachronous HGD or gastric cancer than did the LGD group (HR in the HGD group, 5.23; 95% CI, 1.04–26.24; *P* = 0.044) (Fig. 4B).

## Discussion

In this study, we investigated whether there is a difference in the risk of MGNs after ESD between the LGD group and the HGD group. In the LGD group, the risk of MGNs after ESD was not different than that in the HGD group. In addition, there was also no difference in the risk of metachronous HGD or gastric cancer between the two groups. However, in patients without *H. pylori* infection, the risk of metachronous HGD or gastric cancer was significantly increased in patients with HGD compared to patients with LGD.

In patients with EGC who underwent ESD, several long-term outcome studies reported that the annual incidences of metachronous gastric cancer after ER in EGCs were approximately 3–4%^20–22^. However, few studies reported long-term outcomes after ESD of gastric dysplasia, mainly compared to outcomes after ESD of EGC^15,16^. Yoon et al showed that incidences of metachronous gastric cancer and gastric neoplasm after ER were not different between the LGD group and the EGC group^15^. In this study, 30% of patients in the EGC group were patients with HGD (Vienna classification category 4.1), although the long-term outcomes after ESD might be different between HGD and EGC. Cho *et al.* reported that patients with HGD had a comparable incidence of metachronous gastric cancer after ER during a median follow-up of 42 months compared to patients with EGC^16^. However, differences in outcomes after ESD with respect to histologic types of gastric dysplasia (LGD vs. HGD) have not yet been well studied.

A previous study reported that the risk of progression to gastric cancer was greater in patients with severe dysplasia (HR, 40.14; 95% CI, 32.2–50.1) than in those with mild-to-moderate dysplasia (HR, 3.93; 95% CI, 3.2–4.8) when compared to atrophic gastritis^6^. Although the reported progression rates varied in different studies, patients with HGD had higher rates of progression to invasive carcinoma (10%–68.8%) than those with LGD (2.8–11.5%)^3–7^. A recent study reported that HGD was a significant risk factor for the development of metachronous gastric cancer after ESD of gastric dysplasia (odds ratio, 2.74; *P* = 0.023 vs. LGD)^12^. Similarly, in the present study, the risks of MGNs (HR, 1.45; *P* = 0.425), and metachronous HGD or gastric cancer (HR, 2.42; *P* = 0.214) were increased in the HGD group compared with the LGD group; however, there were no statistical significances between the two groups. Half of MGNs in the HGD group were HGDs or EGCs, and the annual incidence of MGNs was approximately 2.6% (26.1 cases/1,000 person-years) after ESD of HGD, which was a rate similar to that of the annual metachronous gastric cancer incidence in EGC patients who underwent ER^20–22^. Thus, more meticulous follow-up endoscopic evaluations may be needed to detect MGNs in patients who underwent ER for HGD.

A recent randomized controlled trial reported that *H. pylori* treatment significantly reduced metachronous gastric cancer in patients who underwent ER for EGC or HGD^20^. However, in this study, the incidence of metachronous gastric cancer was approximately 1% per year (9.1 cases/1,000 person-years) in patients with successful *H. pylori* eradication^20^. A cohort study also showed approximately 3% annual incidence (29.9 cases/1,000 person-years) of metachronous gastric cancer after ER for EGC in *H. pylori* eradicated patients^23^. These data suggest that long-term endoscopic surveillance after ER might be needed in patients with EGC even after successful *H. pylori* eradication. However, the effects of *H. pylori* eradication on the prevention of MGNs were not consistent for patients who underwent ER for gastric dysplasia^13,14,24^. Two studies showed significantly lower rates of metachronous neoplasm after *H. pylori* eradication^13,24^; meanwhile, another study found no association between *H. pylori* eradication and development of metachronous neoplasm after ER in patients with gastric dysplasia^14^. We found that the risk of metachronous HGD or gastric cancer significantly increased after ESD of HGD in patients without *H. pylori* infection during follow-up (HR, 5.23; *P* = 0.044). The annual incidence of metachronous HGD or gastric cancer was 3.1% (31.3 cases/1,000 person-years) in the HGD group, compared to approximately 0.6% (5.6 cases/1,000 person-years) in the LGD group. Although further well-designed, prospective studies are needed to evaluate the effect of *H. pylori* treatment on the prevention of MGNs, our data suggest that long-term surveillance endoscopy after ER is also needed, even in patients with HGD without *H. pylori* infection.

For patients with gastric dysplasia, the British guideline recommended follow-up endoscopy at 6 months and 12 months after the ER of HGD, and at 12 months after the ER of LGD^11^. As per the European guideline, surveillance endoscopy was recommended in patients with gastric dysplasia in the absence of endoscopically defined lesions, 1 year for LGD, and 6 months to 1-year intervals for HGD^10^. Despite comparable incidences of MGNs after ER of gastric dysplasia, criteria for surveillance with endoscopy after ER for gastric dysplasia have not been established. Thus, long-term surveillance endoscopy after ER of gastric dysplasia should focus on high-risk patients to increase cost-effectiveness. Risk factors associated with metachronous neoplasm or EGC after ER for gastric dysplasia were male sex^12^, old age^14,16^, open-type atrophic gastritis^15^, presence of intestinal metaplasia^12,14^, and HGD^12^. However, the reported risk factors were numerous and inconsistent among different studies. In the present study, risk factors associated with MGNs after ER in patients with gastric dysplasia were not found. In addition, background atrophy and intestinal metaplasia were not a significant risk factor for MGNs development, although HRs were increased. Further studies will be needed to select high-risk patients who undergo long-term surveillance endoscopy after ER of gastric dysplasia.

Our study had several limitations. First, selection bias could not be avoided due to the retrospective study design. Only patients who had follow-up periods greater than 1 year were included. Second, the proportion of patients in the HGD group was only one-fourth of those in the LGD group. Third, we could not assess the association between extent of atrophy and intestinal metaplasia (Operative Link on Gastritis Assessment [OLGA] and Operative Link on Gastric Intestinal Metaplasia [OLGIM]) and risk of gastric dysplasia or cancer. A meta-analysis and cohort studies reported significantly higher gastric cancer risk among high-risk subjects with OLGA or OLGIM stage III/IV^25–27^. Therefore, the European guideline recommended assessment of OLGA and OLGIM stages to identify subjects with high-risk of progression to gastric cancer^10^. However, we evaluated only background atrophy and intestinal metaplasia because we did not perform multiple biopsies for the OLGA and OLGIM stage assessment. Finally, the median follow-period was only 2.5 years. Thus, to evaluate long-term outcomes after ER of gastric dysplasia, more long-term follow-up studies are needed.

In conclusion, the risks of MGNs and metachronous HGD or gastric cancer after ESD were not different between patients with LGD and those with HGD, despite an increased risk noted in the HGD group. However, in patients with negative *H. pylori* infection, the HGD group had significantly increased risk of metachronous HGD or gastric cancer compared to the LGD group. Thus, after ESD of gastric dysplasia, meticulous surveillance endoscopy is needed to detect MGNs, especially in patients with HGD, including those without *H. pylori* infection.

## Supplementary information

Supplementary table 1.
