# Supplementary material for: Risk of metachronous gastric neoplasm occurrence during intermediate-term follow-up period after endoscopic submucosal dissection for gastric dysplasia
Source: Sci Rep. 2020 Apr 21;10:6747. doi: 10.1038/s41598-020-63722-0 (PMC7174371; doi:10.1038/s41598-020-63722-0)
Supplement: Supplementary file 1 — Supplementary table 1. [file 41598_2020_63722_MOESM1_ESM.docx]

**SUPPLEMENTARY INFORMATION**

**Risk of metachronous gastric neoplasm occurrence during intermediate-term follow-up periods after endoscopic submucosal dissection for gastric dysplasia**

Young-Il Kim,^1,2^ Jae Yong Park,^3^ Beom Jin Kim,^3^ Hye Won Hwang,^4^ Soon Auck Hong,^4^ Jae Gyu Kim.^3^

*^1^Center for Gastric -Cancer, National Cancer Center, Goyang, Korea*

*^2^Graduate School of Medicine, Chung-Ang University, Seoul, Korea*

*^3^Department of Internal Medicine, Chung-Ang University College of Medicine, Seoul, Korea*

*^4^Department of Pathology, Chung-Ang University College of Medicine, Seoul, Korea*

**Corresponding Author**: Jae Gyu Kim, M.D., PhD., Department of Internal Medicine, Chung-Ang University College of Medicine, 102 Heukseok-ro, Dongjak-gu, Seoul 06973, Republic of Korea, Tel: +82-2-6299-3147, Fax: +82-2-749-9150, E-mail: jgkimd@cau.ac.kr

ORCID Number 0000-0002-4841-9404

Supplementary table 1. Risk factors for metachronous high-grade dysplasia or gastric cancer*

|  | Univariate analysis† | | *P* |
| --- | --- | --- | --- |
|  | HR | 95% CI |  |
| Age, years | 1.07 | 0.99-1.16 | 0.098 |
| Sex |  |  |  |
| Female | 1.00 |  |  |
| Male | 2.18 | 0.45-10.57 | 0.331 |
| Smoking |  |  |  |
| No | 1.00 |  |  |
| Yes | 0.39 | 0.05-3.22 | 0.322 |
| Alcohol |  |  |  |
| No | 1.00 |  |  |
| Yes | 0.45 | 0.09-2.19 | 0.323 |
| Antiplatelet drug use |  |  |  |
| No | 1.00 |  |  |
| Yes | 1.26 | 0.25-6.25 | 0.779 |
| *H.pylori* status at last follow-up |  |  |  |
| Negative | 1.00 |  |  |
| Positive | 4.81 | 0.55-41.40 | 0.153 |
| Histologic type of dysplasia |  |  |  |
| Low-grade dysplasia | 1.00 |  |  |
| High-grade dysplasia | 2.42 | 0.60-9.73 | 0.214 |
| Tumor size |  |  |  |
| < 1.5 cm | 1.00 |  |  |
| ≥ 1.5 cm | 1.90 | 0.50-7.19 | 0.343 |
| Tumor location |  |  |  |
| Lower third | 1.00 |  |  |
| Middle third | 0.48 | 0.06-4.02 | 0.502 |
| Upper third | 1.90 | 0.23-15.82 | 0.555 |
| Initial multiple lesions |  |  |  |
| No | 1.00 |  |  |
| Yes | 1.24 | 0.15-10.36 | 0.844 |
| Background atrophy |  |  |  |
| Absent | 1.00 |  |  |
| Mild | 4.20 | 0.17-103.68 | 0.381 |
| Moderate to marked | 6.35 | 0.28-143.53 | 0.245 |
| Background intestinal metaplasia |  |  |  |
| Absent | 1.00 |  |  |
| Mild | 0.64 | 0.02-21.26 | 0.803 |
| Moderate to marked | 1.91 | 0.08-42.96 | 0.684 |
| HR, hazard ratio; CI, confidential interval.  *Advanced neoplasms included high-grade dysplasia and gastric cancer.  †The Cox-proportional hazard regression model was used. | | | |
